# 

**Published:** 1843-04

**Authors:** 


### WESTERN JOURNAL

OF

### MEDICINE AND SURGERY.

### EDITORS.

DANIEL DRAKE, M. D.,

PROFESSOR OF PATHOLOGICAL ANATOMY AND CLINICAL MEDICINE
IN THE MEDICAL INSTITUTE OF LOUISVILLE:

LUNSFORD P. YANDELL, M. D.,

PROFESSOR OF CHEMISTRY AND PHARMACY IN THE SAME:

THOMAS W. COLESCOTT, M. D.

### RECEIPTS OF THE MEDICAL JOURNAL FOR MARCH.

| | | |
|---|---|---|
| J. H. Temple, Delta, Miss., | - - - - - | $2 50 |
| Dr. G. W. Foreman, High Grove, Ky., | - - - | 5 00 |
| T. J. Henderson, Richmond, Ala., | - - - | 5 00 |
| J. F. Perkay, Eagle, Ohio, | - - - - | 5 00 |
| B. H. Kerrick, Jefferson County, Ky., | - - | 5 00 |
| Jno. P. Hill, Mt. Sterling, Ala., (discount $2) | - | 5 00 |
| J. B. Coleman, Union Springs, Ala, (discount $1 50,) | | 5 00 |
| J. Templeton, Xenia, Ohio, | - - - - | 11 00 |
| C. M. Godfrey, Kalida, Ohio, | - - - - | 3 00 |
| H. G. Duerson, Westport, Ky. | . - - - | 5 00 |
| —— Adams, North Middletown, Ky., | - - | 5 00 |
| L. A. Sayre, New York, N. Y., | - - - - | 5 00 |
| R. L. Buck, Cold Spring, Miss., | - - - | 5 00 |
| A. C. McLaughlin, Tremont, Ohio, | - - - | 5 00 |
| M. L. Travis, Camden, Tenn., | - - - - | 5 00 |
| J. B. Nash, Dixon, Ill. | - - - - - | 5 00 |
| R. H. Smith, Clay Village, Ky., | - - - | 5 00 |
| J. C. Ross, Clark's Mills, Ark., | - - - - | 10 00 |
| —— Allison, Friendsville, Ill., (discount 63 cents,) | | 6 00 |
| W. J. McDowell, Portsmouth, O,, | - - - | 5 00 |
| W. P. Richardson, Jamestown, Mo. | - - - | 5 00 |

